# Amygdala-prefrontal pathways and the dopamine system affect nociceptive responses in the prefrontal cortex

**DOI:** 10.1186/1471-2202-12-115

**Published:** 2011-11-15

**Authors:** Kitaro Onozawa, Yuki Yagasaki, Yumi Izawa, Hiroyuki Abe, Yoriko Kawakami

**Affiliations:** 1 Department of Oral and Maxillofacial Surgery, Tokyo Women's Medical University Medical Center East, 2-1-10 Nishiogu, Arakawa-ku, 116-8567, Japan; 2 Department of Physiology, School of Medicine, Tokyo Women's Medical University, 8-1 Kawada-cho, Shinjuku-ku, Tokyo 162-8666, Japan

## Abstract

**Background:**

We previously demonstrated nociceptive discharges to be evoked by mechanical noxious stimulation in the prefrontal cortex (PFC). The nociceptive responses recorded in the PFC are conceivably involved in the affective rather than the sensory-discriminative dimension of pain. The PFC receives dense projection from the limbic system. Monosynaptic projections from the basolateral nucleus of the amygdala (BLA) to the PFC are known to produce long-lasting synaptic plasticity. We examined effects of high frequency stimulation (HFS) delivered to the BLA on nociceptive responses in the rat PFC.

**Results:**

HFS induced long lasting suppression (LLS) of the specific high threshold responses of nociceptive neurons in the PFC. Microinjection of N-methyl-D-aspartic acid (NMDA) receptor antagonists (2-amino-5-phosphonovaleric acid (APV), dizocilpine (MK-801)) and also metabotropic glutamate receptor (mGluR) group antagonists (α-methyl-4-carboxyphenylglycine (MCPG), and 2-[(1S,2S)-2-carboxycyclopropyl]-3-(9H-xanthen-9-yl)-D-alanine (LY341495)), prevented the induction of LLS of nociceptive responses. We also examined modulatory effects of dopamine (DA) on the LLS of nociceptive responses. With depletion of DA in response to 6-hydroxydopamine (6-OHDA) injection into the ipsilateral forebrain bundle, LLS of nociceptive responses was decreased, while nociceptive responses were normally evoked. Antagonists of DA receptor subtypes D2 (sulpiride) and D4 (3-{[4-(4-chlorophenyl) piperazin-1-yl] methyl}-1H-pyrrolo [2, 3-b] pyridine (L-745,870)), microinjected into the PFC, inhibited LLS of nociceptive responses.

**Conclusions:**

Our results indicate that BLA-PFC pathways inhibited PFC nociceptive cell activities and that the DA system modifies the BLA-PFC regulatory function.

## Background

Psychological conditions like attention [1] and hypnotic effects [2] exert powerful influences on human pain sensation [3]. The anterior cingulate cortex (ACC) is an area responsible for the affectional dimension of pain [4]. Noxious stimulation applied to peripheral tissues evoked nociceptive responses in rodent cingulate areas [5,6] lesions of which impaired place avoidance test while pain behavior on formalin test was normal [7]. The PFC has crucial roles in conscious pain but not the sensory-discriminative aspect of pain. The psychological modulatory influence on pain response in the PFC could be related to dense projections from the limbic areas [8,9]. Influences of direct projections from the amygdala to the PFC on neural activities have been less investigated electrophysiologically. We analyzed how the projections alter the PFC neural discharges evoked by noxious stimulation.

Neurons of the BLA project directly to the PFC [10], and terminate in layers II and V of the PFC [11]. These projecting neurons from the BLA, forming monosynaptic connections to PFC neurons, are glutamate immuno-positive [12]. BLA-PFC projections using glutamate as a neurotransmitter have the potential to induce plastic changes in cortical synapses. In fact, HFS delivered to the amygdala induced long-term potentiation of field potentials in the PFC [13]. Electrophysiological studies employing PFC slices showed HFS to induce long-term depression (LTD) [14]. BLA-PFC pathways may thus change long-lasting neuronal activities of PFC neurons.

DA, which is considered to regulate attention in the PFC [15], acts as a modulator and induces bidirectional excitatory or inhibitory effects on PFC neurons [16]. DA also modifies plastic changes in PFC neurons of superficial layers [17]. Microinjections of DA into the ACC reduced autotomy scores in a sciatic neurotomy model, indicating DA in the PFC to block long-term nociceptive responses [18]. DA in the PFC may thus modulate pain processing.

Our experiments elucidated that the BLA-PFC projections and the mesocortical DA system affected discharges of PFC neurons elicited by noxious stimulation. The BLA-PFC pathways, and influences of the DA system on these pathways, may underlie psychological states mediating pain sensations.

## Results

### Nociceptive responses recorded in the PFC

**Figure 1:**
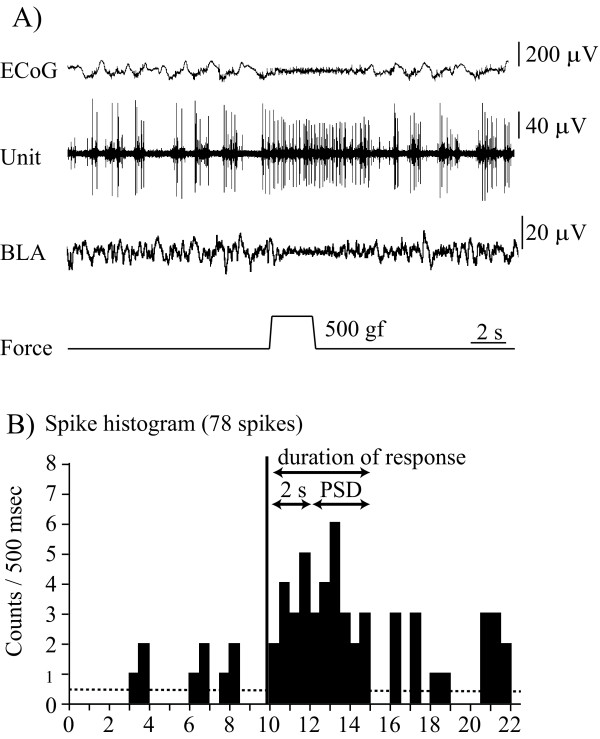
**A typical example of nociceptive discharges evoked by peripheral mechanical stimulation**. **A**. The top trace represents an ECoG. The second trace is multiple unit discharges evoked by mechanical stimulation. The third trace is an EEG recorded via stimulating electrodes in the BLA. Nociceptive stimulation also induced EEG changes. The bottom trace represents a pressure curve. **B**. Durations of responses and post-stimulus discharges (PSD) are shown as a histogram. One bin is 500 msec. Vertical lines represent the stimulus starting point and the horizontal broken line represents the mean level of spontaneous discharges.

Mechanical stimulation induced excitatory responses in PFC neurons, with discharges of a specific high threshold type which persisted during and frequently after stimulation [6]. The spontaneous background discharges of these neurons usually showed the spindle bursts characteristic of urethane anesthesia. Electrocorticograms (ECoG) changed from slow waves with spindle bursts to low amplitude fast waves induced by mechanical stimulation (Figure 1A). The nociceptive responses were recorded without adaptation, if mechanical stimulation was applied every 90 s. We also recorded electroencephalographic (EEG) data in the amygdala, which showed changes in responses to mechanical simulation similar to those in the PFC (Figure 1A).

### HFS delivered to the BLA modified pain responses recorded in the PFC

**Figure 2:**
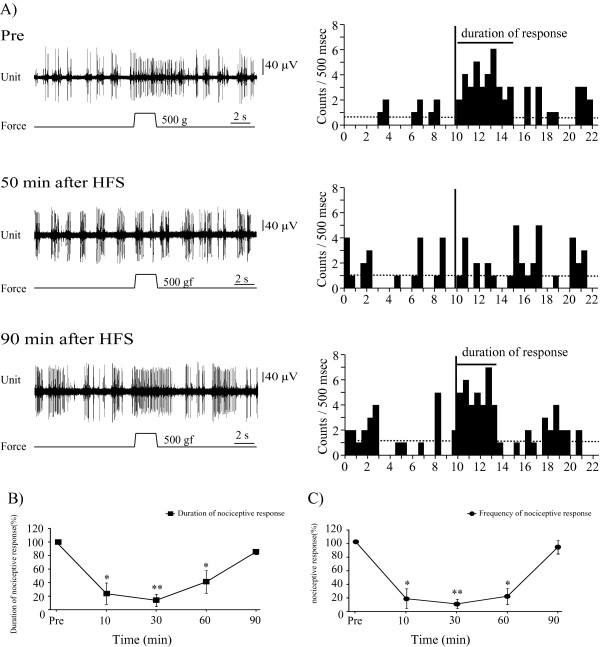
**HFS delivered to the BLA suppressed nociceptive responses recorded in the PFC**. On the left, unit discharges: on the right, the histogram of single unit discharges calculated from the unit discharge. **A**. Unit discharges recorded in the PFC. Pre: Nociceptive responses evoked by 500 gf stimulation applied to the rat tail. At 50 min after HFS delivery to the BLA, the nociceptive responses were entirely blocked. At 90 min after HFS, nociceptive responses had recovered to the pre-HFS control level. **B**. Inhibitory effects of nociceptive responses were induced by HFS delivered to the BLA. The inhibitory effects of nociceptive responses appeared within 10 min and lasted longer than 60 min after HFS. The duration of the control nociceptive responses was assessed as 100% and changes in duration after HFS to the BLA were converted into percentages of the control value. n = 9. *p < 0.05, versus the pre-HFS control value, **p < 0.001, versus the pre-HFS control value. Error bars represent S.E. **C**. The mean discharge frequency of the control nociceptive responses was assessed as 100% and changes in mean discharge frequency after HFS to the BLA were converted into percentages of this control value. *p < 0.05, versus the pre-HFS control value, **p < 0.001, versus the pre-HFS control value. Error bars represent S.E. Mean discharge frequency of nociceptive responses also decreased after HFS in the same manner as the response duration. The statistical significance of differences between results obtained by the two methods was calculated using ANOVA. There were no significant differences between the mean discharge frequency of responses changes (n = 9) and response duration changes (n = 9).

HFS delivered to the BLA impaired PFC nociceptive responses evoked by mechanical stimulation applied to the rat tail (Figure 2A, n = 9, 9 units/9 rats). LLS of nociceptive responses appeared within 10 min and lasted more than 60 min after HFS. Decreases in responses were to 23.6 ± 15.5% of the pre-HFS control level at 10 min (p < 0.05), 13.5 ± 8.9% at 30 min (p < 0.01), 40.8 ± 16.6% at 60 min (p < 0.05) and 85.3 ± 3.7% at 90 min (p = 0.11) (Figure 2B). The mean discharge frequency of responses also decreased after HFS in the same manner as response duration (Figure 2C, p < 0.001). Therefore, we used discharge duration to represent nociceptive responses in this study. Nociceptive responses recovered to pre-HFS levels in approximately 90-120 min. Microinjection of artificial cerebrospinal fluid (ACSF) had no effects on control nociceptive responses in the PFC (n = 5, 5 units/5 rats). HFS delivered to the BLA clearly inhibited nociceptive responses in rats injected with ACSF. At 10 and 30min after HFS, nociceptive responses were significantly suppressed. In the intact group nociceptive responses had not recovered to the pre-stimulus level at 60 min (Figure 2B, C). In contrast, the ACSF group responses recovered to 75.6 ± 25.0% of the pre-HFS level (P = 0.5).

### NMDA receptor blockers impaired LLS of nociceptive responses

**Figure 3:**
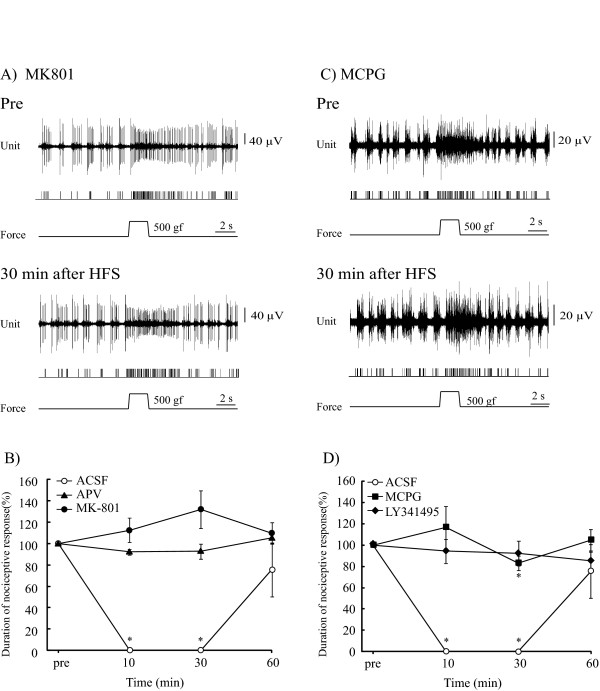
**A glutamate receptor blocker blunted the inhibitory effects of nociceptive responses induced by HFS**. **A**. Pre: Smaller unit discharges in response to mechanical stimulation. The second trace represents single unit responses selected by cluster analysis from multiple units in the top line. At 30 min after HFS, microinjection of MK-801 completely inhibited LLS of nociceptive responses. **B**. NMDA antagonists, APV and MK-801, suppressed the effects of HFS on nociceptive responses. The duration of control nociceptive responses was assessed as 100% and changes in duration after HFS to the BLA were converted into percentages of the control value. There were no significant changes from the pre-HFS control value. In the ACSF group, nociceptive responses were completely blocked at 10 and 30 min after HFS but had recovered to the pre-HFS control value by 60 min. *p < 0.05, versus the pre-HFS control value. Error bars represent S.E. n = 15. **C**. After microinjection of MCPG (an mGluR group I and II antagonist) into the PFC, unit discharges were recorded in the PFC. Pre: prior to HFS delivery to the BLA. Multiple unit discharges responded to peripheral noxious stimulation. At 30 min after HFS, microinjection of MCPG blocked induction of LLS of nociceptive responses. **D**. Effects of mGluR antagonists on LLS of nociceptive response. MCPG partially blocked LLS of nociceptive responses induced by HFS to the BLA. Inhibitory effects were observed only at 30 min after HFS. An mGluR group II antagonist, LY341495, completely blocked LLS of nociceptive responses. *p < 0.05, versus the pre-HFS control value. Error bars represent S.E. n = 12.

NMDA receptor blockers impaired effects of HFS on nociceptive responses. MK-801, which had no effects on control nociceptive responses, completely inhibited the LLS of nociceptive responses induced by HFS delivered to the BLA (n = 5, 5 units/5 rats). Rates of changes in nociceptive responses were 112.6 ± 11.5% of the pre-HFS value at 10 min (p = 0.27), 132.0 ± 17.8% at 30 min (p = 0.14) and 109.6 ± 10.1% at 60 min (P = 0.35) (Figure 3A, B). As with MK-801, microinjection of APV also reduced the effects of HFS on nociceptive responses (n = 5, 5 units/5 rats, Figure 3B). There were no significant differences in rates of changes versus the control value; 92.1 ± 2.8% at 10 min (p = 0.08), 92.8 ± 7.0% at 30 min (p = 0.35) and 105.3 ± 5.0% at 60 min (p = 0.29).

### Metabotropic glutamate receptor blocker

MCPG, an mGluR group I and II antagonist, partially impaired LLS of nociceptive responses induced by HFS delivered to the BLA (n = 5, 5 units/5 rats). Durations of nociceptive responses did not differ significantly from the control level at 10 min (p = 0.50, 116.8 ± 19.8%), or at 60 min (104.8 ±10.1%, p = 0.08). Inhibitory effects were, in fact, observed only at 30 min after HFS (83.1 ± 6.8%, versus pre-HFS control level, p < 0.05) (Figure 3C, D). However, the nociceptive response at 30 min was significantly longer than that of the ACSF group (0%, p < 0.05). A selective antagonist of group II mGluR, LY341495, completely blocked LLS of nociceptive responses induced by HFS (n = 7, 7 units/6 rats). Durations of pain responses were 94.4 ± 11.4% at 10 min (p = 0.24), 92.4 ±11.6% at 30 min (p = 0.61) and 85.3 ± 8.4% at 60 min (p = 0.09) (Figure 3D).

### DA depletion impaired LLS of nociceptive responses

**Figure 4:**
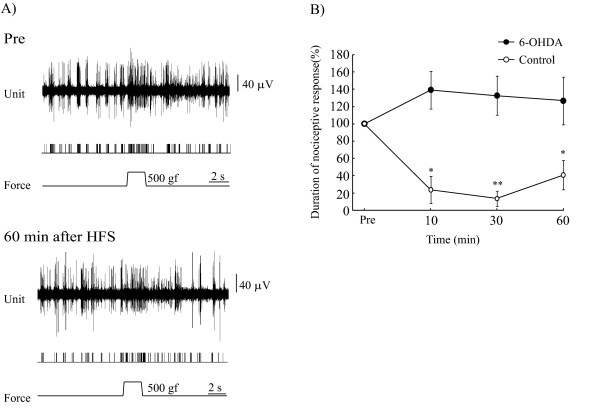
**DA depletion impaired the inhibitory effects of nociceptive responses induced by HFS**. **A**. Nociceptive discharges were recorded in the PFC of the side ipsilateral to 6-OHDA injection. Pre: Nociceptive responses were normally evoked by mechanical stimulation delivered to the rat tail. At 60 min after HFS delivery to the BLA, there were no apparent effects on nociceptive responses. DA depletion completely blocked LLS of nociceptive responses induced by HFS. **B**. The duration of nociceptive responses tended to increase in this group, but the difference from the pre-HFS control value did not reach statistical significance. Error bars represent S.E. n = 8 The control figure is same as figure 2 B. *p < 0.05, **p < 0.001, versus the pre-HFS control value.

DA depletion was established by apomorphine tests three weeks after 6-OHDA injection into the medial forebrain bundle (MFB, n = 8, 8 units/8 rats). In apomorphine test-positive animals, background ECoG showed low voltage fast waves, as described in a previous report [19]. However, nociceptive responses were normally evoked by mechanical stimulation delivered to the tail. There was no difference in mean nociceptive responses (p = 0.15) between the control and 6-OHDA groups (4.6 s and 3.6 s, respectively). HFS applied to the BLA had no effects on the nociceptive responses in this group (Figure 4A). Rates of changes in nociceptive responses were 139.3 ± 21.8% at 10 min (p = 0.12), 132.7 ± 22.5% at 30 min (p = 0.16) and 126.7 ± 27.7% at 60 min (p = 0.26) after HFS (Figure 4B). The duration of nociceptive responses tended to increase in DA-depleted animals, but the difference versus the pre-HFS control value did not reach statistical significance.

### DA receptor subtype antagonists

**Figure 5:**
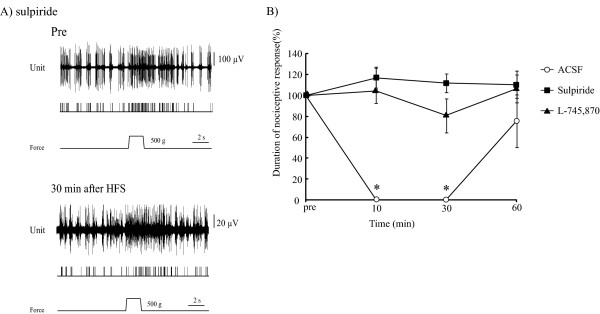
**DA receptor subtypes modified LLS of nociceptive responses induced by HFS**. **A**. Pre: A typical case with sulpiride injection is shown. Nociceptive responses were normally evoked by mechanical stimulation delivered to the rat tail after microinjection of sulpiride (upper trace). At 30 min after HFS, a D2 receptor antagonist completely blocked LLS of nociceptive responses induced by HFS to the BLA (lower trace). **B**. D2 and D4 receptor antagonists suppressed the induction of LLS of nociceptive responses by HFS. Error bars represent S.E. n = 19. The control figure is same as figure 3 B, C. *p < 0.05, **p < 0.001, versus the pre-HFS control value.

A D2 receptor antagonist, sulpiride (n = 5, 5 units/4 rats), was also injected into the PFC. Sulpiride significantly blocked the inhibitory effects of HFS to the BLA (Figure 5A, B). Durations of pain responses were 116.8 ± 9.4% at 10 min (p = 0.14), 111.8 ± 8.9% at 30 min (p = 0.47) and 110.4 ±1 3.1% at 60 min (p = 0.69) after HFS to the BLA. In the sulpiride-treated group, the durations of pain responses tended to be increased but did not differ significantly from the pre-HFS control value (Figure 5B).

A specific D4 receptor antagonist, L-745, 870 (n = 14, 14 units/8 rats), also significantly blocked the LLS of nociceptive responses induced by HFS without changes in either nociceptive responses or background ECoG. Durations of pain responses were 104.3 ± 11.5% at 10 min (p = 0.73, versus pre HFS value), 80.9 ± 16.27% at 30 min (p = 0.27) and 106.4 ± 13.1% at 60 min (p = 0.55) after HFS to the BLA (Figure 5B). These results indicate that L-745,870 blocks LLS of nociceptive responses evoked by HFS applied to the BLA.

## Discussion

We previously recorded nociceptive responses in layer II and also found Fos expression to be induced by mechanical noxious stimulation in surface layers of the PFC [6]. Monosynaptic projections from the BLA also terminated in layer II of the PFC [11]. Assuming that nociceptive information from peripheral nociceptors and inputs from the BLA integrate at the same pyramidal cells in layer II of the PFC, HFS delivered to BLA-PFC may induce heterosynaptic plasticity. Heterosynaptic-LTD, which has been demonstrated in perforant path synapses of the hippocampus [20,21], is reportedly blocked by NMDA receptor blockers [22]. In our experiments, APV and MK-801 impaired LLS of nociceptive responses. NMDA receptor activities were required for the induction of LLS of nociceptive responses, suggesting heterosynaptic-LTD like mechanisms to underlie these responses. Brief application of Group I and II mGluR agonists [23] or co-activation of mGluR II and NMDA receptors reportedly induces LTD in pyramidal neurons [24]. Depolarization of postsynaptic cells is indispensable for mGluR mediated heterosynaptic-LTD [25]. In our experiments, glutamate release via ascending pathways relying sensory information and NMDA activation by HFS delivered to the BLA produced LLS of nociceptive responses in the PFC characterizing the responses as LTD.

The DA system has been assumed to modulate PFC plasticity [26]. Co-application of DA and the mGluR agonist without tetanic stimulation induced LTD in PFC pyramidal neurons [27]. DA does not directly mediate synaptic transmission but affects it by altering the synaptic properties of target neurons [15]. A behavioral study of pain demonstrated that DA depletion induces significant changes in thresholds for mechanical noxious stimuli [28]. Partial DA depletion in our experiments had no effects on nociceptive responses themselves but blocked plastic changes in nociceptive responses. DA application produces only a small membrane depolarization (2-3 mV) in pyramidal cells [29] but affects spike afterpotentials [30] which consist of after-hyperpolarization or depolarization. Partial depletion of DA may inhibit slow after-depolarization followed by LLS induction, while having little effect on the initiation of action potentials [31]. Emotional influences of inputs from the BLA on PFC nociceptive responses might be modulated by DA.

A D2 receptor antagonist, sulpiride, blocked LLS of nociceptive responses in the PFC. Postsynaptic D2 receptor activities reduce membrane depolarization evoked by a-amino-3-hydroxy-5-methyl-4-isoxazolepropionic acid agonist application [15]. Moreover, D2 receptor knockout mice showed impaired LTD induction in response to tetanic stimulation delivered to cortico-striatal pathways [32]. In addition to postsynaptic D2 receptor inhibitory effects, presynaptic D2 receptors showed impaired glutamate release in the ventral tegmental area [33]. D2 antagonists may affect both pre- and post-synaptic receptors and thereby block the induction of LLS of nociceptive responses. D4 receptors, which are highly expressed in PFC pyramidal cells [34], inhibit adenylate cyclase through Gi/o-proteins by functioning as D2-like receptors [35]. Electrophysiological experiments using current and voltage clamp methods demonstrated spontaneous hyper-excitability of pyramidal neurons in a D4 receptor knockout mouse model [36]. Hence, D4 receptors normally exert an inhibitory influence on the activities of pyramidal neurons. D4 receptor activities may facilitate induction of LLS of nociceptive responses by HFS delivered to the BLA.

According to human brain image analysis, the strength of conscious pain reflects activities of the ACC [2,37,38]. BLA-PFC projections may transmit aversive information from the limbic area to the PFC, which is involved in psychological states of dependent pain sensation like fear conditioning analgesia. BLA-PFC pathways are reportedly involved in fear expression and extinction [39]. NMDA activation in the BLA and PFC are required for relearning of inhibitory fear responses in extinction [40]. Our LLS results suggest that impulses from the BLA depressed nociceptive responses, as a consequence, pain recognition in the PFC was inhibited in fear responses.

The mesocortical DA system from the ventral tegmental area to the PFC plays roles in psychological conditions such as attention and motivation. Our results indicate that amygdala inputs to the PFC influence pain sensations via emotional stimulation and that the DA system exerts modulatory effects on conscious pain processing.

## Conclusions

Mechanical noxious stimulation applied to peripheral tissue evoked nociceptive discharges in the PFC. HFS delivered to the BLA inhibited nociceptive responses, which are involved in the mGluR-mediated LTD-like mechanism. This inhibition was impaired by DA receptor subtype (D2 and D4) blockers. BLA-PFC projections influence pain responses and the DA system exerts modulatory effects on pain responses recorded in the PFC. Inputs from the BLA and DA system to the PFC may be responsible for conscious pain.

## Methods

### Animal preparation

Male Wistar rats (300-400 g; Sankyo Laboratory Co., Tokyo, Japan) were used in all experiments. The rats were housed under controlled temperature (25°C) and humidity (40-45%) conditions with a 12-h light/dark cycle, and had free access to food and water. Experiments conformed to the guidelines issued by the National Institutes of Health for Laboratory Animals. All procedures were performed in accordance with the guidelines for animal care of the Animal Experiments Committee of Tokyo Women's Medical University and have been approved by Ethical Review Committee of Animal Experiments Tokyo Women's Medical University (Serial No.021). Efforts were made to minimize the number of animals used and their suffering.

### Recording and Stimulating electrodes

**Figure 6:**
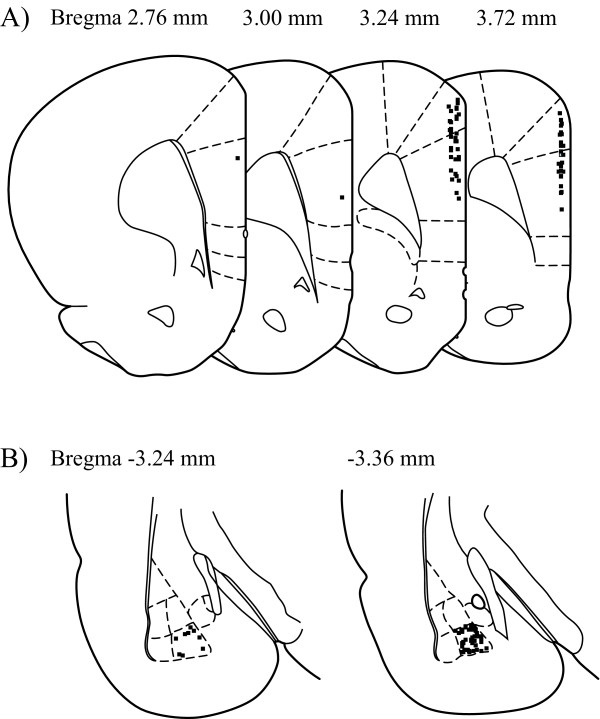
**Areas of mPFC recording and BLA stimulation**. **A**. Recording points of unit discharges. The solid squares represent actual recording points. All unit discharges are scattered throughout surface layers of the cingulate and prelimbic areas. The numbers above represent distance from the bregma. **B**. In the BLA, the solid squares represent stimulus points. The numbers represent distance from the bregma.

All animals were anesthetized with urethane (1.0-1.2 g/kg, i.p) and placed in a stereotaxic frame (Narishige, Tokyo, Japan). Tungsten microelectrodes (Frederick Haer & Co., Bowdon, ME, USA) with an impedance of 9-12 MΩ implanted in the BLA as stimulus electrodes were also used as recording electrodes. HFS (100 Hz, +20 μA for 30 s) was delivered to the BLA. We recorded extracellular unit discharges and local field ECoG from a typical nociceptive specific neuron through the same recording electrode [6]. The unit spikes were processed with a multichannel amplifier (MEG-6100; 0.08-3000 Hz; Nihon Kohden Co., Tokyo, Japan) and an active filter (DV-04; 500-3000 Hz; NF Electronic Instruments Co., Yokohama, Japan). Through a memory oscilloscope (VC-11; Nihon Kohden), the data were fed into a thermal array recorder (Nihon-Kohden) for paper recording and a personal computer (Macintosh G4; Apple Co., Tokyo, Japan) via an integrated system (PowerLab/4SP; Mountain View, CA, USA) for recording storage and later off-line analysis. In all animals, the recording electrodes were positioned in the medial PFC (mPFC, coordinates in mm: 2.7-3.7 anterior and 0.1-0.5 lateral to the bregma) (Figure 6A). As shown in Figure 6B, the stimulating electrodes were positioned in the BLA (coordinates in mm: 3.2-3.4 posterior and 4.4-4.5 lateral to the bregma, 7.75-7.8 ventrally below the dura).

### Mechanical stimulation

We applied mechanical pressure to the tail, at 1.0-4.0 cm distal to the body, via a mechanical stimulator (DPS-270; DIA Medical System Co., Tokyo, Japan), using a probe with a circular contact area with a 1 mm in diameter tip. Mechanical stimuli were delivered every 90 s at constant force with a feedback system. Stimulus intensities used in this experiment were 500 gf with a 0.1 s rising time to maximum force and a 2 s hold time. Previous studies showed a nociceptive stimulus intensity of 300 gf to be sufficient to induce C-fiber mediated activity in peripheral nerves [41]. We employed a typical nociceptive specific neuron, which responded only to a stimulus intensity exceeding 300gf [6].

### Drugs and injection techniques

In the drug injection experiments, we used recording electrodes with a Teflon microtube (TF205-074, Unique Medical Co., Japan). Each drug was injected into the mPFC using a microinjector (55-1111, Harvard Apparatus Co., Miami, FL, USA). Extracellular unit discharges and local ECoG were recorded for at least 15 min before and after the injection to ensure that the drug injections had no effects on nociceptive responses in the PFC. We also injected ACSF (4 μl, pH = 7.4) alone at a rate of 20 μl/hr as a control.

Drugs used in the extracellular unit recording were MK-801 (1.0 μg/4 μl, NMDA receptor antagonist; Sigma, St. Louis, MO, USA) [42], APV (20 ng/10 μl, NMDA receptor antagonist; Sigma) [43], MCPG (1.0 μg/3.5 μl, mGluR group I and II antagonist; Sigma) [44], LY341495 (0.4 μg/3.2 μl, mGluR group II antagonist; Tocris Cookson, Ellisville, MO, USA) [45], sulpiride (17 ng/5 μl, DA D2 receptor antagonist; Sigma) [46] and L-745,870 (0.4 μg/2 μl, DA D4 receptor antagonist; Sigma) [47] at a rate of 20 μl/hr. These drugs were dissolved in ACSF alone.

### Data analysis

A single unit spike was discriminated on the basis of the height and width of each unit from a multiple unit recording by applying cluster analysis methods with the software program "Chart and Spike Histogram" (AD Instruments Co., Tokyo, Japan). Each bin of histograms consisted of spikes during a 500 ms period. The duration of the responses and discharge frequency both reflect stimulus intensity [6]. Thereby, we measured both duration and mean discharge frequency as responses evoked by mechanical stimulation. The durations of evoked tonic discharges exceeding mean spontaneous discharges on the histogram were assessed as the responses durations (Figure 1B). The mean value of three successive nociceptive responses just before HFS was assessed as 100% i.e. as the control. We recorded ECoG and spontaneous discharge as baseline data for 10 s before applying mechanical pressure to the tail. Spontaneous mean discharge frequency and ECoG did not change after as compared to before mechanical stimulation and HFS. However, we waited for recovery until the same ECoG pattern or administered additional anesthesia to maintain the same ECoG pattern.

### Unilateral injection of 6-OHDA

Rats were anesthetized with Nembutal (50 mg/kg i.p). Then, 5 μl of 6-OHDA HCl (2 mg in 1ml of saline containing 0.1% ascorbic acid; Sigma) were injected into the left MFB (coordinates in mm: 4.5 posterior and 1.1 lateral to the bregma, 8.2 ventrally below the dura) through a cannula with a microinjection pump at a rate of 20 μl/hr, and the cannula was left in place for 10 min after the completion of pumping. At the end of the injection, the skin was sutured. We assessed motor disturbances 3 weeks after 6-OHDA treatments by observing full-rotations in a cylindrical container (40 cm diameter, LE 902/Rp Container; Panlabs.I., Barcelona, Spain) for 30 min after apomorphine (1 mg/kg i.p.) administration. The same experimental procedures as described above (Recording and Stimulating electrodes) were carried out after DA depletion had been established. Nociceptive discharges were recorded in the PFC ipsilateral to the 6-OHDA injection. As a control, no apomorphine-induced rotational asymmetry was observed in the intact group.

### Statistical analysis

The significance of differences in discharges evoked by mechanical stimuli was assessed with the nonparametric paired-test (Wilcoxon) to compare pre- and post-HFS values (Stat View-J5.0; SAS Institute Inc., Berkeley, CA, USA). Differences in nociceptive responses between the drug-injected groups and the ACSF-injected group were statistically analyzed with repeated measure analysis of variance (ANOVA). Data are expressed as means ± standard errors (S.E.). A probability level <0.05 was considered significant.

### Histological site of unit recordings

The locations of units were marked with a positive electric current lesion (direct current, +80 μA for 15 s). At the end of each experiment, the animals were perfused with normal saline and 4% paraformaldehyde. The brains were removed, sectioned (50 μm) and then stained with hematoxylin-eosin solution to examine the recording sites under light microscopy. Only successive penetrations, located in the cingulate or the prelimbic area, were used for data analysis.

## List of abbreviations

(ACC): anterior cingulate cortex; (ACSF): artificial cerebrospinal fluid; (ANOVA): repeated measure analysis of variance; (APV): 2-amino-5-phosphonovaleric acid; (BLA): basolateral nucleus of the amygdala; (DA): dopamine; (MK-801): dizocilpine; (ECoG): electrocorticogram; (EEG): electroencephalogram; 3-{[4-(4-chlorophenyl) piperazin-1-yl] methyl}-1H-pyrrolo [2,3-b] pyridine (L-745,870); (HFS): high frequency stimulation; (LLS): long-lasting suppression; (LTD): long-term depression; (LY341495): 2-[(1S,2S)-2-carboxycyclopropyl]-3-(9H-xanthen-9-yl)-D-alanine; (MCPG): α-methyl-4-carboxyphenylglycine; (MFB): medial forebrain bundle; (mGluR): metabotropic glutamate receptor, (mPFC): medial prefrontal cortex; (NMDA): N-methyl-D-aspartic acid; (6-OHDA): 6-hydroxydopamine; (PFC): prefrontal cortex; (PSD): post-stimulus discharges; (S.E.): standard error.

## Authors' contributions

OK, YY and YI carried out the experiments. OK and HA analyzed the experiments. OK wrote the manuscript. YK conceived the study, designed experiments and helped to draft the manuscript. All authors read and approved the final manuscript.
